# Effects of phenolic acids and quercetin-3-*O*-rutinoside on the bitterness and astringency of green tea infusion

**DOI:** 10.1038/s41538-022-00124-8

**Published:** 2022-01-27

**Authors:** Yu-Hong Chen, Yan-Hong Zhang, Gen-Sheng Chen, Jun-Feng Yin, Jian-Xin Chen, Fang Wang, Yong-Quan Xu

**Affiliations:** 1 Tea Research Institute Chinese Academy of Agricultural Sciences, Key Laboratory of Tea Biology and Resources Utilization, Ministry of Agriculture, 9 South Meiling Road, Hangzhou, 310008 China; 2 Graduate School of Chinese Academy of Agricultural Sciences, Beijing, 100081 China

**Keywords:** Agriculture, Biochemistry

## Abstract

Phenolic acids are important taste components in green tea. The aim of this study was to analyze the taste characteristics of phenolic acids and their influence on the bitterness and astringency of green tea by sensory evaluation and chemical determination. The major tea phenolic acids and quercetin-3-*O*-rutinoside (Que-rut) were significantly positively correlated with the bitterness (*r* = 0.757, *p* < 0.01; *r* = 0.605, *p* < 0.05) and astringency (*r* = 0.870, *p* < 0.01; *r* = 0.576, *p* < 0.05) of green tea infusion. The phenolic acids have a sour and astringent taste, whereas Que-rut has a mild astringent taste. Phenolic acids and Que-rut can increase the bitterness of epigallocatechin gallate (EGCG). However, these components behaved differently for astringency on EGCG. Gallic acid (GA) enhances the astringency throughout all the concentrations in this study. While it seemed to be double effects of caffeic acid (CA), chlorogenic acid (CGA), and Que-rut on that, i.e., the inhibition at lower concentrations (CA: 0–0.2 mM; CGA: 0–0.2 mM; Que-rut: 0–0.05 mM) but enhancement at higher ones. The phenolic acids and Que-rut interacted synergistically with tea infusion and as their concentration increased, the synergistic enhancement of the bitterness and astringency of tea infusion increased. These findings help provide a theoretical basis for improving the taste of middle and green tea.

## Introduction

Green tea is one of the most widely consumed drinks worldwide, especially in China, which accounts for 60% of total consumption. In 2019, China’s green tea consumption exceeded 1.2 million tons^1^. Green tea is made from the new leaves or buds of the tea tree, without fermentation, through curing, shaping, and drying. It retains most of the natural phytochemicals from the fresh leaves, including polyphenols, caffeine, amino acids, and vitamins. Green tea is popular with many consumers due to its various perceived health benefits, e.g., anticancer, antioxidant, anti-inflammatory, anti-diabetic, and anti-obesity^2–6^, and complex taste characteristics comprised of bitterness, astringency, umami, and sweet aftertaste.

Bitterness and astringency are extremely important sensory attributes of green tea infusion^7^, and their intensities strongly influence the desirability and consumption of green tea and related products. Tea polyphenols are very important taste substances in tea infusion, including catechins, flavonols, proanthocyanidins, and phenolic acids^8^, which mainly affect the bitter and astringent taste^9^. Catechins account for 70–80% of the total polyphenols, including galloylated catechins and non-galloylated catechins^10^. The concentrations of galloylated catechins, such as epicatechin-3-gallate (ECG), are highly correlated with the bitterness and astringency of green tea infusions^11^. Phenolic acids are a type of tea polyphenols and account for about 5% of the dry weight of fresh tea leaves. Typical phenolic acids, such as gallic acid (GA), chlorogenic acid (CGA), and caffeic acid (CA), impart a sour and astringent taste, which increases with the phenolic acid concentration^12,13^. The concentration of GA and CGA is positively correlated with the bitterness and astringency of white tea, and GA has the biggest influence on the astringency of white tea^14^. Flavonol glycosides, such as quercetin-3-*O*-rutinoside (Que-rut), also make a significant contribution to the taste of tea^15,16^. Que-rut imparts a velvety soft astringency and is the main contributor to the astringent taste perceived from black tea^17^.

In addition, green tea infusion is a mixed system consisting of polyphenols, amino acids, purine alkaloids, sugars, etc., and have taste interactions between these substances. Epigallocatechin gallate (EGCG) was found to synergistically enhance the bitterness and astringency of caffeine, and inhibit the sweetness of sucrose^18^. GA has been found to lower the astringency of alum^19^. Que-rut can increase the bitterness of caffeine^20^. However, little research has been reported on the sensory evaluation of phenolic acids and Que-rut, and the effects of phenolic acids and Que-rut on the bitterness and astringency of green tea infusion are still unclear.

Further research is needed to elucidate the relationships between phenolic acids, Que-rut, and green tea taste. The aim of this study was to investigate the contribution of phenolic acids and Que-rut to the bitterness and astringency of green tea infusion. The findings will help understand the relationship between the polyphenol compositions and taste of green tea and facilitate the taste improvement of tea and tea-based beverages.

## Results and discussion

### Identification of key phenolic compounds contributing to the bitterness and astringency of green tea infusions

To identify the key phenolic compounds that contribute to the bitterness and astringency of green tea infusions, the bitterness and astringency of tea samples were scored by a standard sensory evaluation method. The intensities of the bitterness of the tea infusions ranged from 3.00 ± 0.07 to 6.03 ± 0.04, and the average intensity was 4.59. The intensities of the astringency of the tea infusions ranged from 2.95 ± 0.07 to 6.00 ± 0.00, and the average astringency was 4.58 (Supplementary Table 1). Moreover, a borderline significant relationship was found between bitterness and astringency (*r* = 0.931, *p* < 0.01). This result is consistent with previous reports^11,21^.

Polyphenols were reported to be the major contributors to the bitterness and astringency of green tea infusions^9,22,23^. To clarify the influence of different polyphenolic components on bitterness and astringency, the concentrations of major polyphenols in 16 representative tea samples were determined, including total tea polyphenols, eight catechins, 12 flavonols, and three phenolic acids (Supplementary Table 2–5). After combining the intensity of bitterness and astringency of the tea infusions with the concentrations of total and individual polyphenols, the correlations between them were analyzed (Table 1). Total tea polyphenols, catechins, flavanols, and phenolic acids, and the concentrations of 11 individual polyphenolic compounds (most catechins, kaempferol (Kae), Que-rut, vitexin-2-*O*-rhamnoside (Vit-rha), and GA) positively correlated with bitterness intensity were identified. Total tea polyphenols, catechins, flavonols, and phenolic acids, and the concentrations of 14 individual polyphenolic compounds (eight catechins, Kae, kaempferol-3-*O*-rutinoside (Kae-rut), Que-rut, Vit-rha, CGA, and GA) positively correlated with astringency intensity were also identified.

**Table 1:** Correlation coefficients between the intensity of bitterness and astringency, and polyphenols in green tea infusion.

Polyphenols	Bitterness intensity	Astringence intensity
Total tea polyphenols	0.947**	0.938**
C	0.719**	0.661**
GC	0.705**	0.668**
CG	0.762**	0.695**
GCG	0.774**	0.746**
EC	0.536*	0.649**
EGC	0.450	0.558*
ECG	0.820**	0.853**
EGCG	0.922**	0.895**
Total catechins	0.881**	0.904**
Kae	0.734**	0.803**
Kae-rut	0.431	0.498**
Kae-glu	0.478	0.351
Que	−0.137	−0.144
Que-rha	0.130	0.302
Que-rut	0.605*	0.576*
Que-glu	0.249	0.381
Que-gala	−0.331	−0.405
Myr-rha	0.276	0.371
Myr-gala	0.391	0.429
Vit	0.204	0.323
Vit-rha	0.708**	0.651**
Total flavonoids	0.534*	0.651**
CA	−0.388	−0.309
CGA	0.441	0.644**
GA	0.671**	0.634**
Total phenolic acids	0.757**	0.870**

*C* (+)-catechin, *GC* (+)-gallocatechin, *CG* (-)-catechin-3-gallate, *GCG* (-)-gallocatechin-3-gallate, *EC* (−)-epicatechin, *EGC* (−)-epigallocatechin, *ECG* (-)-epicatechin-3-gallate, *EGCG* (−)-epigallocatechin gallate, *Kae* kaempferol, *Kae-rut* kaempferol-3-*O*-rutinoside, *Kae-glu* kaempferol-3-*O*-glucoside, *Que* quercetin, *Que-rha* quercetin-3-*O*-rhamnoside, *Que-rut* quercetin-3-*O*-rutin, *Que-glu* quercetin-3-*O*-glucoside, *Que-gala* quercetin-3-*O*-galactoside, *Myr-rha* myricetin-3-*O*-rhamnoside, *Myr-gala* myricetin-3-*O*-galactoside, *Vit* vitexin, *Vit-rha* vitexin-2-*O*-rhamnoside, *CA* caffic acid, *CGA* chlorogenic acid, *GA* gallic acid.

*means *p* < 0.05 and **means *p* < 0.01.

Based on these correlations, the contribution ratios of each component to the bitterness and astringency of the 16 green tea infusions were calculated as Dot (Dose-over-threshold) factors (Table 2). The Dot factor depends on both the taste intensity of a compound and its concentration. A Dot factor >1, means that the compound makes a significant contribution to the taste of the tea infusion^15,24^ and the greater the Dot factor, the greater the contribution^25^. The main contributors to the bitterness of tea infusion were the catechins epicatechin-3-gallate (ECG) and EGCG. The main contributors to the astringency of tea infusion were the catechins such as epigallocatechin (EGC), ECG and EGCG, and the flavonols including Kae-rut, kaempferol-3-*O*-glucoside (Kae-glu), Que-rut, quercetin-3-*O*-glucoside (Que-glu), quercetin-3-*O*-galactoside (Que-gala), and Myr-gala. Of these, the flavonols were the major contributors to astringency. The Dot factor of Que-rut was the highest, with an average value of 4113.99 (Table 2). GA, CGA, CA all have strong sour and astringent tastes, but their Dot factors were very low because of their low concentrations in the tea infusion, particularly CA. Based on the data in Tables 1 and 2, the foregoing results indicated that phenolic acids and Que-rut had a significant positive relationship with the bitterness and astringency of tea infusions, which were also the major contributors to the astringency of green tea infusion besides catechins.

**Table 2:** Dot analysis of the polyphenols in green tea infusion.

Polyphenols	Threshold (µmol/L)	Min	Max	Average
Group 1: Bitter compounds				
C	860^a^	0.02	0.09	0.05
GC	1630^a^	0.05	0.20	0.10
CG	170^a^	0.01	0.04	0.02
GCG	330^a^	0.03	0.21	0.10
EC	860^a^	0.13	0.47	0.30
EGC	1630^a^	0.13	0.75	0.40
ECG	180^a^	0.77	2.80	1.77
EGCG	220^a^	2.23	7.50	5.09
Group 2: Astringent compounds				
C	690^a^	0.02	0.12	0.06
GC	330^a^	0.27	1.00	0.50
CG	115^a^	0.01	0.06	0.03
GCG	220^a^	0.04	0.31	0.14
EC	860^a^	0.13	0.47	0.30
EGC	260^a^	0.79	4.69	2.54
ECG	135^a^	1.03	3.73	2.35
EGCG	160^a^	3.06	10.32	6.99
Kae-rut	0.25^b^	11.75	156.88	51.89
Kae-glu	0.67^b^	0.77	4.27	1.96
Que-rut	0.00115^b^	1010.33	10577.65	4113.99
Que-glu	0.65^b^	1.74	12.25	4.88
Que-gala	0.43^b^	2.33	8.48	4.61
Myr-rha	10.50^b^	0.08	1.69	0.47
Myr-gala	2.70^b^	0.31	3.07	1.23
CA	72^c^	0.0001	0.0003	0.0002
CGA	50^c^	0.01	0.64	0.22
GA	200^c^	0.11	0.47	0.22

^a^Thresholds were obtained from the literature^7^.

^b^Thresholds were obtained from the literature^14^.

^c^Thresholds were obtained from the literature^12^.

### Concentration-taste intensity curves of three phenolic acids and Que-rut in green tea

**Fig. 1:**
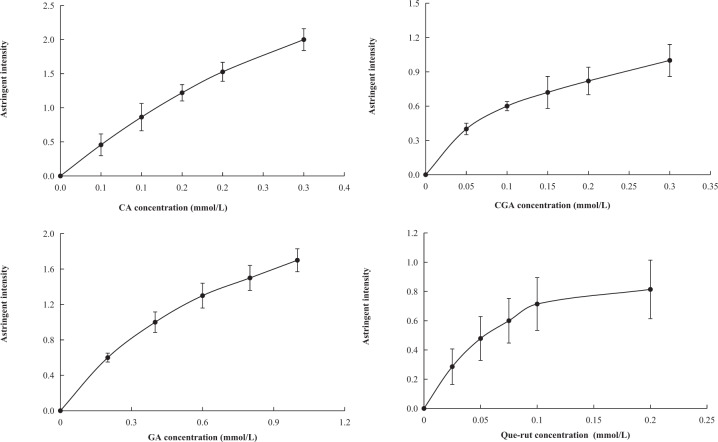
Concentration-astringent intensity curves of the three phenolic acids and Que-rut. CA caffeic acid, CGA chlorogenic acid, GA gallic acid, Que-rut quercetin-3-O-rutinoside. Data are means (±SD) of three replicates.

The concentration-taste intensity curves (Fig. 1) of the three phenolic acids and Que-rut were constructed. The phenolic acids in tea account for about 5% of the dry weight of fresh leaves, of which GA is about 0.5 to 1.4% (w/w), CGA is about 0.3%, and the concentration of CA is very low^8^. The concentration of Que-rut in green tea infusion is 0.02–0.08 mmol/L. In this study, 0–0.3 mmol/L solutions of CA and CGA, 0–1.0 mmol/L solutions of GA, and 0–0.2 mmol/L solutions of Que-rut were sensorially evaluated for bitterness and astringency, over a range of concentrations. CA, CGA, and GA had negligible bitter tastes but were sour and astringent. Que-rut had a mild astringent taste. At 0.2 mmol/L, the astringency followed the order CA > CGA > Que-rut > GA (Fig. 1), but these compounds had low astringency scores. The astringency concentration–intensity curves of phenolic acids and Que-rut conformed to the S-shape of the psychophysical curve^26,27^, and the curves were good fits to cubic functions, with *R*^2^ values greater than 0.998 (Table 3). The results showed that the cubic function models were reliable and accurate.

**Table 3:** Functions used for the phenolic acids and Que-rut concentration-astringent intensity curves.

Components	Cubic functions^a^	*R*^2^
CA	y = 2.2222x^3^ − 10.857x^2^ + 9.7373x − 0.0038	0.9995
CGA	y = 66.667x^3^ − 40.19x^2^ + 9.3762x + 0.0062	0.9988
GA	y = 1.1574x^3^ − 2.9861x^2^ + 3.5271x + 0.0008	0.9999
Que-rut	y = 128.27x^3^ − 68.058x^2^ + 12.534x + 0.0041	0.9991

^a^y is the taste intensity, and x is the concentration of each compound (mmol/L).

### Effects of phenolic acids and Que-rut on the bitterness and astringency of EGCG solution

**Fig. 2:**
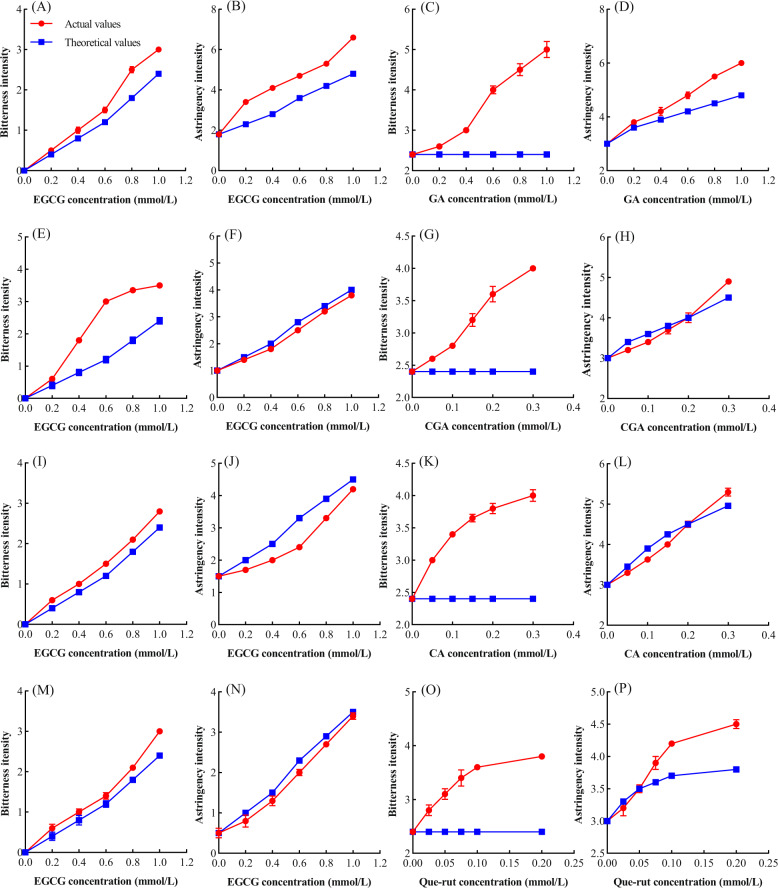
Taste interactions between EGCG, and typical phenolic acids and Que-rut. Bitter and astringent taste interactions of EGCG at different concentrations with GA (**A**, **B**), CGA (**E**, **F**), CA (**I**, **J**), Que-rut (**M**, **N**). Bitter and astringent taste interactions of GA (**C**, **D**), CGA (**G**, **H**), CA (**K**, **L**), Que-rut (**O**, **P**) at different concentrations with EGCG. Actual values represent mixed system sensory scores between the different concentrations of EGCG and GA, CGA, CA, and Que-rut. Theoretical values represent the sum of the bitter and astringent taste points of each concentration of EGCG and the theoretical bitter and astringent taste of GA, CGA, CA, and Que-rut.

An interactive effect between different taste compounds has been reported^28^. Different taste compounds may contribute to the same, or different taste attributes. When the taste substances are mixed, they may have synergistic, additive, or inhibitory interactions. EGCG is the most abundant catechin in tea and has both a bitter and astringent taste. Phenolic acids and Que-rut have similar astringency taste attributes to EGCG through sensory evaluation. When studying the effects of substances with the same taste attributes, the taste intensity of the mixture should be compared with the sum of the intensities of each individual component to identify interaction effects. If the actual value of the mixture is less than the sum of the taste theoretical values of each individual component, it is an inhibitory effect between these components, and the opposite is synergistic interaction. In this study, the taste interactions between EGCG, phenolic acids, and Que-rut which have similar astringency taste were analyzed (Fig. 2).

The bitterness of EGCG solution was significantly enhanced by 1 mmol/L GA, 0.2 mmol/L CGA, 0.2 mmol/L CA, and 0.05 mmol/L Que-rut (Fig. 2A, E, I, M). The bitterness of EGCG solution increased in a concentration-dependent manner with the phenolic acids and Que-rut (Fig. 2C, G, K, O). The reports on astringency interactions found that the increase in the astringency of EGCG by 0.2 mmol/L CGA, 0.2 mmol/L CA, and 0.05 mmol/L Que-rut was significantly lower than that of EGCG with the same astringency intensity, whereas that of 1 mmol/L GA was higher than that of EGCG (Fig. 2B, F, J, N). The effect of different concentrations of phenolic acids and Que-rut on the 1 mmol/L EGCG solution was further investigated (Fig. 2D, H, L, P). At 0.2 mmol/L CGA, 0.2 mmol/L CA, and 0.05 mmol/L Que-rut, the astringency interactions had a turning point. At 0–0.2 mmol/L CGA or CA, and at 0–0.05 mmol/L Que-rut, there was a synergy for bitterness and inhibition for astringency with EGCG. At concentrations of CGA, or CA above 0.2 mmol/L, or at a concentration of Que-rut above 0.05 mmol/L, there was a positive synergy for bitterness and astringency with EGCG. There was also a clear positive synergy for bitterness and astringency between GA and EGCG, and the effect increased with GA concentration. A previous study found a positive association between bitterness and astringency^21^; phenolic acids and Que-rut with low bitterness enhance the bitterness of EGCG, which may be related to their higher astringency.

### Effects of phenolic acids and Que-rut on the bitterness and astringency of green tea infusion

**Fig. 3:**
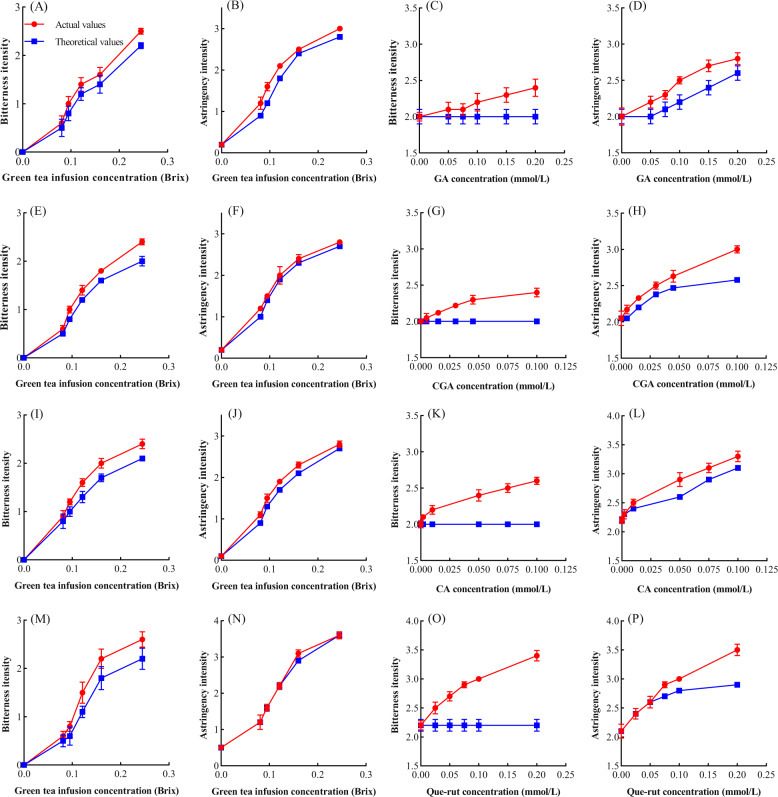
Taste interactions between green tea infusion and typical phenolic acids and Que-rut. GA (**A**, **B**), CGA (**E**, **F**), CA (**I**, **J**), and Que-rut (**M**, **N**) show the bitter and astringent taste interactions at different concentrations of green tea infusion. GA (**C**, **D**), CGA (**G**, **H**), CA (**K**, **L**), Que-rut (**O**, **P**) show the bitter and astringent taste interactions with green tea infusion at different concentrations of GA, CGA, CA, and Que-rut. Actual values represent mixed system sensory scores between the different concentrations of green tea infusion and GA, CGA, CA, and Que-rut. Theoretical values represent the sum of the bitter and astringent taste points of each concentration of green tea infusion and the theoretical bitter and astringent taste of GA, CGA, CA, and Que-rut.

The taste of green tea infusion mainly arises from polyphenols, caffeine, amino acids, organic acids, and carbohydrates^29^. The catechins that impart bitterness and astringency have been systematically studied; and the bitterness of green tea was highly correlated with the concentration of EGCG and ECG, however, the astringency is less correlated with catechins concentration, but increased GA concentration can strengthen the astringency of tea infusion^11,18^. The effect of phenolic acids and Que-rut on the taste of green tea infusion was studied, because the intensity of bitterness and astringency were significantly correlated (Table 3) with the concentrations of total phenolic acids (*r* = 0.757, *r* = 0.870, *p* < 0.01) and Que-rut (*r* = 0.605, *r* = 0.576, *p* < 0.05). GA, CGA, and CA enhanced the bitterness and astringency of green tea infusion and had synergistic effects. Que-rut, at concentrations of 0.00–0.05 mmol/L and the combination of Que-rut and green tea infusion had a synergistic effect on bitterness and a superimposed effect on astringency. At Que-rut concentrations above 0.05 mmol/L, the synergistic increase in bitterness of the tea infusion was greater and there was a synergistic effect on astringency. Furthermore, the synergistic effects increased significantly with the increasing concentrations (Fig. 3). The increase in astringency in tea infusions may arise from increased concentrations of phenolic acids and rutin themselves (Fig. 1), from the synergistic effects with EGCG (Fig. 2), or from the interactions with other substances. These findings are also consistent with previous studies^11,30^. The astringency of tea infusion cannot be well quantified using only the catechins concentration. Phenolic acids and Que-rut, especially GA and Que-rut, made a greater contribution to the astringency of tea infusion.

In addition, as can be found from Fig. 2, a low concentration of CA, CGA, and Que-rut inhibit the astringency of EGCG. In contrast, the situation is just the opposite in green tea infusion as shown from Fig. 3. It is possible that phenolic acids and Que-rut may have an effect of taste interaction with other substances, and greater than the interaction with EGCG may be responsible for such a discrepant result. Alternatively, the sensitivity of the mouth to taste substances increases in the mixed system is a likely cause for these results. Previous studies have shown that for interactions between multivariate mixtures, the individual monomer threshold would decrease in the mixture system^31^. Based on these results of the study, we believe that the interaction between multiple similar or non-similar substances is worth continuing, thus exploring the impact of all kinds of taste substances on the taste of tea infusions.

The present study showed that the concentrations of gallic acid, chlorogenic acid, caffeic acid, and Que-rut were significantly positively correlated with the intensities of bitterness and astringency in green tea infusion. Phenolic acids and Que-rut are astringent, but not significantly bitter. Phenolic acids and Que-rut can increase the bitterness of epigallocatechin gallate (EGCG). However, these components behaved differently for astringency on EGCG. Phenolic acids and Que-rut can enhance both the bitterness and astringency of green tea infusion. The synergistic effects increased significantly with increasing concentrations of phenolic acids and Que-rut. Whether phenolic acids and Que-rut interact with substances besides EGCG and leading to the improved bitterness and astringency of tea infusion deserves to explore and perfect further. These findings could be of great value for perfecting the chemical theory of green tea taste and enabling the systematic improvement of the taste of green tea infusion.

## Methods

### Chemicals

Standard catechins (catechin (C), gallocatechin (GC), catechin-3-gallate (CG), gallocatechin-3-gallate (GCG), epicatechin (EC), epigallocatechin (EGC), epicatechin-3-gallate (ECG), epigallocatechin gallate (EGCG)), flavonols (kaempferol (Kae), kaempferol-3-*O*-rutinoside (Kae-rut), kaempferol-3-*O*-glucoside (Kae-glu), quercetin (Que), quercetin-3-*O*-rhamnoside (Que-rha), quercetin-3-*O*-rutin (Que-rut), quercetin-3-*O*-glucoside (Que-glu), quercetin-3-*O*-galactoside (Que-gala), myricetin-3-*O*-rhamnoside (Myr-rha), myricetin-3-*O*-galactoside (Myr-gala), vitexin (Vit), vitexin-2-*O*-rhamnoside (Vit-rha)) and phenolic acids (gallic acid (GA), chlorogenic acid (CGA), caffeic acid (CA)) were from Sigma-Aldrich China-Mainland (Shanghai, China). Analytical reagents (ferrous sulfate, potassium sodium tartrate, disodium hydrogen phosphate, potassium dihydrogen phosphate, acetic acid) were from Ningbo Chemical Reagent Co., Ltd. (Ningbo, China). Chromatography-grade reagents (methanol, formic acid, acetonitrile) were bought from Shanghai Aladdin Biochemical Technology Co., Ltd. (Shanghai, China). Pure water was from Hangzhou Wahaha Group Co., Ltd. (Hangzhou, China) and was used for all the experiments. The pH of the pure water was 6.82 ± 0.03, and its electrical conductivity was 1.84 ± 0.13 μS/cm.

### Tea samples and preparation of infusions

A total of 16 green tea samples were harvested from the National Tea Tree Resource Nursery (Hangzhou, China) in 2017. The tender shoots with one leaf and a bud were processed into tea samples after fixing, rolling, and drying according to the traditional baking process. Each sample (3.0 g) was brewed with boiling water (150 mL) for 4 min, following the latest standard tea sensory evaluation method (GB/T 23776-2018)^32^. After the tea infusion had cooled to room temperature (25 °C), its bitterness and astringency were scored by a taste panel and the chemical compositions were analyzed.

### Sensory evaluation

The sensory evaluation procedure for phenolic acid solutions, Que-rut solutions, and green tea infusions was based on a previously reported method for catechin solutions and green tea infusions^11^. The sensory panel was composed of five trained panelists, three men and two women, aged from 25 to 48, and all from the Tea Research Institute of the Chinese Academy of Agricultural Sciences (Hangzhou, China). The five trained panelists were conducted in accordance with the principles set forth in the Declaration of Helsinki and informed written consent was obtained. This study was approved by the Zhejiang Gongshang University Human Ethics Committee (Approval No. 2020-12). The scoring process was carried out in a constant temperature and humidity environment (25 °C, relative humidity around 75%). The panelists were trained to evaluate bitterness and astringency with EGCG solutions of different concentrations and assigned different concentrations of EGCG to the corresponding bitterness and astringency intensity scores. The scoring of taste intensity was done on a ten points scale, divided into five intervals^11,18^, i.e., not bitter/astringent (0–2 points), slightly bitter/astringent (2–4 points), bitter/astringent (4–6 points), very bitter/astringent (6–8 points), and extremely bitter/astringent (8–10 points). In the sensory evaluation, a sample solution (50 mL) was prepared in a clear glass 30 min in advance of the panelists performing the sensory evaluation. First, each panelist sipped ~15 mL of the sample solution and swirled it in the mouth for 7–8 s, to evaluate the bitterness, then expelled the solution and evaluated the astringency during the following 3–4 s. Finally, each sample was given an overall score. There was a 5-min interval between the evaluation of each sample and the panelists rinsed their mouths with pure water after each sample evaluation. The evaluations were repeated three times on different days and the results were analyzed statistically.

### Determination of chemical compositions of tea infusions

Total tea polyphenols and total catechins were determined according to the Chinese Standards GB/T 21733-2008^33^ and GB/T 8313-2018^34^, respectively.

Flavonols were determined by HPLC, as described previously, with some modifications^16^. The tea infusion was filtered through a 0.22-μm Millipore filter before analysis. The HPLC was a Shimadzu LC-2010A (Shimadzu Corporation, Kyoto, Japan), fitted with a Symmetry^®^ C18 column (5 μm, 250 × 4.6 mm I.D., Waters, Milford, MA, USA) and an Agilent 1100 series ultraviolet detector (Agilent Corporation, Santa Clara, CA, USA) set at 360 nm. The injection volume was 20 μL, the mobile phase flow rate was 1 mL/min and the column temperature was 25 °C. The mobile phases were different mixtures of acetonitrile/formic acid/water; phase A had a 3:0.15:96.85 ratio and phase B had a 30:0.15:69.85 ratio. The mobile phase elution gradient was 0% B from 0–23 min, ramped linearly to 50% B at 30 min, 62.5% B at 40 min, 80% B at 45 min, 100% B at 48 min, maintained at 100% B until 83 min, then back to 0% B at 85 min.

The concentrations of phenolic acids were determined using UPLC-QE-Orbitrap-MS. according to the previously published method^35^. The tea infusions were filtered through a 0.22-μm Millipore filter before injection (Model Shimadzu LC-2010A; Shimadzu Corporation, Kyoto, Japan). The separation was performed on an ACQUITY UPLC HSS T3 column (1.8 μm, 2.1 mm × 100 mm, Waters, Milford, MA, USA). The gradient separation was carried out using 0.1% formic acid in water and acetonitrile as mobile phases A and B, with the flow rate at 0.3 mL/min for 12 min and the column temperature at 40 °C. The elution gradient under the following conditions: 0–1 min, 5% B; 1–2 min, 5–10% B; 2–6 min, 10–35% B; 6–8.5 min, 35–100% B; 8.5–9.5 min, 100% B; 9.5–10 min, 100–5% B; 10–12 min, 5% B. The Q-Orbitrap mass spectrometer with electrospray ionization (ESI) was used for MS analysis and operated in negative ionization full scan mode. The flow rates of auxiliary gas and sheath gas were 10 and 45 (arbitrary units), respectively. The temperature of the auxiliary gas heater was 300 °C. The capillary temperature was 320 °C. The resolution of full scan and ddMS2 were 70,000 and 35000, respectively. The full MS scan ranges were set from 66.7 to 1000 *m/z*. Using phenolic acid standards to make a corresponding calibration curve for quantification.

### Analysis of taste interactions

#### Analysis of the taste interactions between phenolic acids or Que-rut and EGCG

Five EGCG solutions (0.2, 0.4, 0.6, 0.8, and 1.0 mmol/L) were prepared, and then divided each group of EGCG solutions into two equal parts, one is the control group, and the other is the interaction group. Four solutions were prepared as follows: GA (1.0 mmol/L) with an astringency of 1.8; CGA (0.2 mmol/L) with an astringency of 1.0; CA (0.2 mmol/L) with an astringency of 1.5; Que-rut (0.05 mmol/L) with an astringency of 0.5. These four solutions were combined with each of five EGCG solutions in the interaction group in a 1:1 ratio to generate 20 binary combinations (the concentrations of the above solutions were the concentrations of the substances in the compound solutions). The bitterness and astringency of each group of ten solutions (control group and interaction group) were evaluated by the taste panel, to analyze the taste interactions.

A solution of EGCG (1.0 mmol/L), with a bitterness of 2.4, and an astringency of 3.0, was combined with each of the following solutions: GA (0, 0.2, 0.4, 0.6, 0.8, and 1.0 mmol/L), CGA (0, 0.05, 0.1, 0.15, 0.2, and 0.3 mmol/L), CA (0, 0.05, 0.1, 0.15, 0.2, and 0.3 mmol/L), and Que-rut (0, 0.025, 0.05, 0.075, 0.1, and 0.2 mmol/L). The bitterness and astringency of each group of six solutions (24 solutions in total) were evaluated by the taste panel, to analyze the taste interactions.

#### Analysis of the taste interactions between phenolic acids or Que-rut and green tea infusion

Green tea infusions were brewed with distilled water (1:50 w/v) at 100 °C for 4 min, and then diluted 1:2, 1:3, 1:4, 1:5, and 1:6 (v/v) with distilled water, and then divided each group of tea infusions into two equal parts, one is the control group, and the other is the interaction group. Solutions of GA (0.1 mmol/L, astringency 0.2), CGA (0.015 mmol/L, astringency 0.2), CA (0.002 mmol/L, astringency 0.1), and Que-rut (0.05 mmol/L, astringency 0.5) were mixed with different concentrations of tea infusions in the interaction group. The bitterness and astringency of each group of ten solutions (control group and interaction group) were evaluated by the taste panel, to analyze the taste interactions.

The green tea infusions above were diluted 1:2. The soluble solids concentration of the tea infusion was 0.16 Brix. Solutions of GA (0, 0.05, 0.075, 0.1, 0.15, and 0.2 mmol/L), CGA (0, 0.005, 0.015, 0.03, 0.045, and 0.1 mmol/L), CA (0, 0.002, 0.01, 0.05, 0.075, and 0.1 mmol/L), and Que-rut (0, 0.025, 0.05, 0.075, 0.1, and 0.2 mmol/L) were mixed with the tea infusion. The bitterness and astringency of each group of six solutions were evaluated by the taste panel to analyze the taste interactions.

#### Statistical analysis

All data were presented as the mean ± standard deviation (three replicates). The results were analyzed with SPSS Version 22.0 using one-way ANOVA to demonstrate the significant differences. *P* values < 0.05 were considered statistically significant.

## Supplementary information

Supplementary information

Informed consent form

The Form of Human Sensory Ethical Committee Inspection

## Data Availability

The authors declare that all data generated or analyzed during this study are included in this published article and its supplementary information files. Related data are available from the authors upon reasonable request.
