# Supplementary material for: Effects of phenolic acids and quercetin-3-O-rutinoside on the bitterness and astringency of green tea infusion
Source: NPJ Sci Food. 2022 Jan 27;6:8. doi: 10.1038/s41538-022-00124-8 (PMC8795203; doi:10.1038/s41538-022-00124-8)
Supplement: Supplementary file 1 — Supplementary information [file 41538_2022_124_MOESM1_ESM.pdf]

Effects of phenolic acids and Que-rut

on the bitterness and astringency of green tea

Supplementary table 1

Bitterness and astringency of green tea infusions

Sample	Brand	Bitterness	Astringency
1	Pingyangtezao	3.00±0.07	2.95±0.07
2	Maolv	3.08±0.11	3.05±0.07
3	TR129	3.40±0.14	3.15±0.21
4	Hanlv	3.78±0.04	3.33±0.04
5	Anjibaicha	3.90±0.14	3.50±0.00
6	Shuigucha	3.15±0.21	3.65±0.21
7	Zhongcha NO.102	4.12±0.17	4.05±0.21
8	Lanhai	5.55±0.07	5.05±0.07
9	Fuyun NO.6	4.50±0.00	5.00±0.00
10	Longjingziya	5.00±0.21	5.55±0.07
11	Zhenong NO.138	5.10±0.21	5.50±0.00
12	Gaoqiaojuye	5.23±0.11	5.75±0.07
13	Zhangke NO.1	5.70±0.07	5.00±0.00
14	Changbolv	6.00±0.00	5.83±0.11
15	Liancha NO.2	6.03±0.04	5.90±0.14
16	Dongting NO.301	5.85±0.07	6.00±0.00

Data are means (± SD) of three replicates.

Supplementary table 2

Concentrations of tea polyphenols in green tea infusions (mg/L)

Sample	Tea polyphenols	Sample	Tea polyphenols
1	949.15±2.77	9	2011.80±5.54
2	1201.60±5.54	10	2068.55±2.77
3	1334.67±11.07	11	2207.50±11.07
4	1348.37±8.30	12	2281.86±16.61
5	1438.40±13.84	13	2604.77±2.77
6	1608.65±11.07	14	2626.29±22.14
7	1761.30±11.07	15	2702.62±8.30
8	1972.66±11.07	16	2733.93±8.30

Supplementary table 3

Concentrations of catechins in green tea infusions (mg/L)

Sample	C	GC	CG	GCG	EC	EGC	ECG	EGCG	Total
1	4.90±0.21	31.30±3.09	0.56±0.08	4.18±0.61	56.52±0.57	170.27±1.73	61.31±1.25	224.54±0.25	553.58±6.64
2	5.10±0.05	30.10±0.14	0.41±0.10	5.56±0.79	55.45±0.50	151.45±0.64	74.72±2.34	281.94±0.36	604.73±2.36
3	12.42±0.28	35.14±0.65	1.09±0.15	9.02±1.08	67.58±0.35	194.14±1.46	93.91±0.57	370.51±0.65	783.81±3.19
4	8.53±0.33	27.12±0.16	1.02±0.17	7.85±1.04	53.96±1.17	137.49±1.76	115.46±0.79	373.26±0.32	724.69±2.45
5	7.44±0.22	37.34±0.48	0.36±0.06	6.32±0.97	57.77±0.37	177.83±1.81	69.98±1.07	402.32±0.35	759.35±2.93
6	5.76±0.42	40.26±0.36	0.77±0.11	10.36±1.41	33.25±0.8	88.31±0.44	95.00±2.15	445.01±0.37	718.71±1.27
7	8.39±1.07	43.18±0.26	0.72±0.08	9.25±0.30	57.74±1.94	180.94±1.33	99.94±0.09	433.07±0.10	833.23±1.01
8	14.45±0.29	101.22±1.72	0.88±0.08	9.13±0.54	44.73±2.00	62.85±0.21	146.71±0.83	508.81±1.69	888.80±1.68
9	10.60±0.28	35.35±0.5	0.92±0.19	6.23±1.09	116.16±0.14	246.39±0.68	194.25±2.25	466.67±0.05	1076.57±3.34
10	14.71±0.18	41.10±2.76	1.11±0.16	10.60±0.98	91.30±0.01	283.36±2.01	184.53±0.24	591.12±0.36	1217.83±5.60
11	9.08±0.03	50.77±1.09	0.57±0.06	12.65±0.99	110.41±0.45	200.31±0.43	222.69±1.39	539.68±0.63	1146.16±1.00
12	23.18±0.25	81.99±0.02	2.18±0.05	31.21±0.32	90.47±0.07	312.77±0.32	127.08±2.20	634.13±0.19	1303.01±1.85
13	23.59±0.00	50.51±1.33	2.64±0.18	27.67±0.93	88.12±0.49	233.01±2.34	202.90±0.61	756.57±0.27	1385.02±4.40
14	17.71±0.51	70.78±0.31	2.79±0.09	26.29±1.35	96.61±0.42	276.87±1.63	194.16±0.39	755.91±0.32	1441.12±2.53
15	9.54±0.51	47.00±3.10	2.26±0.18	24.54±1.85	55.99±0.24	141.84±2.57	165.48±1.33	674.09±0.11	1120.74±8.86
16	17.05±0.33	85.96±0.87	3.18±0.15	30.76±0.98	116.31±0.68	373.58±1.14	201.67±0.18	749.99±2.53	1578.51±0.59

Supplementary table 4

Concentration of flavonoids in green tea infusions (mg/L)

Sample	Kae	Kae-rut	Kae-glu	Que	Que-rha	Que-rut	Que-glu	Que-gala	Myr-rha	Myr-gala	Vit	Vit-rha	Total
1	0.48±0.04	3.38±0.13	0.49±0.28	0.17±0.00	0.48±0.09	1.13±0.06	0.68±0.04	0.60±0.24	0.40±0.03	0.40±0.04	0.33±0.00	0.46±0.01	9.02±0.24
2	0.43±0.08	3.16±0.25	0.23±0.13	0.93±0.06	1.03±0.11	1.24±0.10	0.79±0.12	0.96±0.14	1.26±0.72	0.76±0.17	0.16±0.03	0.93±0.02	11.88±0.60
3	0.94±0.02	1.75±0.09	0.64±0.64	1.29±0.04	5.26±0.11	0.52±0.13	1.28±0.29	1.69±0.06	0.47±0.29	1.53±0.17	0.22±0.02	1.05±0.03	16.65±1.51
4	0.31±0.01	3.63±0.01	0.46±0.39	1.09±0.14	0.74±0.01	2.67±0.39	1.14±0.93	0.98±0.02	2.34±0.29	0.88±0.01	0.05±0.01	0.79±0.01	15.07±1.10
5	0.88±0.02	2.44±0.02	0.41±0.13	0.09±0.03	2.48±0.69	2.39±0.06	1.80±1.59	0.98±0.89	2.58±0.38	2.18±0.16	0.09±0.06	0.85±0.05	17.17±1.18
6	0.68±0.05	2.93±1.40	0.69±0.89	0.17±0.12	5.20±0.29	0.71±0.44	2.24±0.67	0.97±0.39	0.37±0.05	1.12±0.10	0.41±0.01	0.59±0.17	16.07±2.44
7	0.95±0.09	2.86±0.30	0.45±0.45	1.20±0.02	5.46±0.31	1.56±0.91	0.52±0.72	1.33±0.10	0.75±0.70	2.69±0.41	0.41±0.01	1.07±0.01	19.26±1.12
8	0.95±0.01	17.54±1.53	0.69±0.85	0.05±0.00	1.78±1.88	4.57±0.37	0.94±0.53	0.47±0.27	1.29±0.27	0.44±0.12	0.01±0.01	1.03±0.03	29.75±2.05
9	1.47±0.01	23.32±0.74	0.28±0.16	0.88±0.02	13.33±0.47	7.43±0.22	2.12±0.17	0.93±0.09	7.36±0.51	0.77±0.05	0.07±0.01	0.80±0.00	58.76±1.79
10	0.97±0.05	3.44±0.14	0.57±0.73	0.52±0.04	7.15±0.03	1.06±0.57	2.06±0.00	0.86±0.65	1.20±0.10	2.49±0.21	0.16±0.13	1.39±0.01	21.87±1.98
11	1.32±0.17	18.41±0.15	0.48±0.38	0.37±0.34	1.02±0.03	3.11±0.15	1.34±0.18	0.60±0.00	8.22±0.31	0.66±0.29	0.33±0.02	1.38±0.04	37.25±0.89
12	1.19±0.07	9.18±0.57	0.86±1.04	0.47±0.24	3.27±0.05	2.46±0.88	1.21±0.14	0.67±0.13	1.24±0.33	2.55±0.23	2.42±0.15	1.30±0.13	26.82±1.97
13	1.00±0.03	3.77±0.31	1.28±1.72	0.41±0.20	1.14±1.04	2.84±0.33	0.95±0.20	1.28±0.85	1.23±0.78	1.58±0.08	0.48±0.05	1.74±0.11	17.72±3.11
14	1.14±0.04	3.94±0.46	0.74±0.70	0.81±0.65	5.08±0.09	3.46±0.65	1.93±0.52	0.62±0.38	2.48±0.33	2.25±0.02	0.35±0.01	1.20±0.02	24.01±0.11
15	1.68±0.07	18.91±0.35	0.65±0.75	0.55±0.62	0.60±0.01	7.17±0.23	0.86±0.42	0.84±0.73	3.58±1.37	1.29±0.19	0.22±0.00	1.05±0.07	37.39±1.87
16	1.23±0.01	4.73±0.37	0.49±0.43	0.77±0.77	10.78±0.13	3.90±1.38	3.70±0.17	0.94±0.23	1.68±1.51	3.98±0.10	0.53±0.07	1.22±0.08	33.95±2.70

Kae, kaempferol; Kae-rut, kaempferol-3-O-rutinoside; Kae-glu, kaempferol-3-O-glucoside; Que, quercetin; Que-rha, quercetin-3-O-rhamnoside; Que-rut, quercetin-3-O-rutin; Que-glu, quercetin-3-O-glucoside; Que-gala, quercetin-3-O-galactoside; Myr-rha, myricetin-3-O-rhamnoside; Myr-gala, myricetin-3-O-galactoside; Vit, vitexin; Vit-rha, vitexin-2-O-rhamnoside

Supplementary table 5

Concentration of phenolic acids in green tea infusions (mg/L)

Tea NO.	Gallic acid	Chlorogenic acid	Caffic acid	Total
1	6.57±0.13	0.65±0.50	0.0018±0.00	7.22±0.37
2	9.00±0.02	0.99±0.54	0.0017±0.00	9.98±0.56
3	7.47±0.23	0.78±0.71	0.0053±0.00	8.25±0.49
4	8.14±0.21	0.17±0.05	0.0031±0.00	8.31±0.15
5	7.37±0.29	1.07±1.16	0.0034±0.00	8.44±1.46
6	11.53±0.92	3.77±1.73	0.0142±0.01	15.31±0.83
7	14.76±0.64	0.61±0.38	0.0015±0.00	15.37±0.26
8	12.55±0.45	2.25±2.81	0.0019±0.00	14.80±2.37
9	10.34±0.08	11.29±13.19	0.0024±0.00	21.63±13.11
10	8.37±0.31	6.67±8.09	0.0023±0.00	15.04±7.79
11	13.13±0.27	5.53±3.64	0.0016±0.00	18.66±3.37
12	10.46±0.17	9.93±10.59	0.0017±0.00	20.39±10.76
13	12.07±0.24	2.35±2.09	0.0028±0.00	14.42±1.85
14	15.27±0.17	9.78±10.81	0.0045±0.00	25.05±10.98
15	21.54±0.26	0.59±0.58	0.0015±0.00	22.14±0.84
16	12.81±0.09	5.82±6.29	0.0009±0.00	18.64±6.38
