# Supplementary material for: Effects of phenolic acids and quercetin-3-O-rutinoside on the bitterness and astringency of green tea infusion
Source: NPJ Sci Food. 2022 Jan 27;6:8. doi: 10.1038/s41538-022-00124-8 (PMC8795203; doi:10.1038/s41538-022-00124-8)
Supplement: Supplementary file 2 — Informed consent form [file 41538_2022_124_MOESM2_ESM.pdf]

Informed consent form

Project Title: Effects of phenolic acids and quercetin-3-O-rutinoside on the bitterness and astringency of green tea infusion

1. Purpose of this Research Study: You have been asked to participate in this study because you are a professional tea sensory reviewer. The aim of this study was to investigate the contribution of phenolic acids and Que-rut to the bitterness and astringency of green tea infusion.

2. Background: Bitterness and astringency are extremely important sensory attributes of green tea infusion, and their intensities strongly influence the desirability and consumption of green tea and related products. Tea polyphenols are very important taste substances in tea infusion, including catechins, flavonols, proanthocyanidins, and phenolic acids, which mainly affect the bitter and astringent taste. There has been research on the bitter and astringent taste of catechins is relatively mature, however, little research has been reported on the sensory evaluation of phenolic acids and Que-rut, and the effects of phenolic acids and Que-rut on the bitterness and astringency of green tea infusion are still unclear.

3. What will be down: You need to score the different monomer substances and tea infusions. The scoring process was carried out in a constant temperature and humidity environment (25°C, relative humidity around 75%). You will be trained to evaluate bitterness and astringency with EGCG solutions of different concentrations and assigned different concentrations of EGCG to the corresponding bitterness and astringency intensity scores. The scoring of taste intensity was done on a 10 points scale, divided into five intervals, i.e., not bitter/astringent (0-2 points), slightly bitter/astringent (2-4 points), bitter/astringent (4-6 points), very bitter/astringent (6-8 points), and extremely bitter/astringent (8-10 points). In the sensory evaluation, sample solution (50 mL) was prepared in a clear glass 30 minutes in advance of the panelists performing the sensory evaluation. First, each panelist sipped ~15 mL of the sample solution and swirled it in the mouth for 7-8 seconds, to evaluate the bitterness, then expelled the solution and evaluated the astringency during the following 3-4 seconds. Finally, each sample was given an overall score. There was a 5-minute interval between the evaluation of each sample and the panelists rinsed their mouths with pure water after each sample evaluation. The evaluations were repeated three times on different days and the results were analyzed statistically.

4. Possible Risks and Discomforts: All samples were of edible grade and did not need to be swallowed into the body and had no side effects on the body.

5. Offer to Answer Questions: If you have any further questions, you can contact the major experimenter Chen yuhong.

6. Signature for Consent: The above-named investigator has answered your questions and you agree to be a research subject in this study. You have carefully read the information and understand fully your rights as a potential subject in a research experiment involving people as subjects.

Subject's Signature: 开泽娟 Date: 2020.12.25

Investigator's Signature: 陈雨鸿 Date: 2020.12.25

Informed consent form

Project Title: Effects of phenolic acids and quercetin-3-O-rutinoside on the bitterness and astringency of green tea infusion

1. Purpose of this Research Study: You have been asked to participate in this study because you are a professional tea sensory reviewer. The aim of this study was to investigate the contribution of phenolic acids and Que-rut to the bitterness and astringency of green tea infusion.

2. Background: Bitterness and astringency are extremely important sensory attributes of green tea infusion, and their intensities strongly influence the desirability and consumption of green tea and related products. Tea polyphenols are very important taste substances in tea infusion, including catechins, flavonols, proanthocyanidins, and phenolic acids, which mainly affect the bitter and astringent taste. There has been research on the bitter and astringent taste of catechins is relatively mature, however, little research has been reported on the sensory evaluation of phenolic acids and Que-rut, and the effects of phenolic acids and Que-rut on the bitterness and astringency of green tea infusion are still unclear.

3. What will be down: You need to score the different monomer substances and tea infusions. The scoring process was carried out in a constant temperature and humidity environment (25°C, relative humidity around 75%). You will be trained to evaluate bitterness and astringency with EGCG solutions of different concentrations and assigned different concentrations of EGCG to the corresponding bitterness and astringency intensity scores. The scoring of taste intensity was done on a 10 points scale, divided into five intervals, i.e., not bitter/astringent (0-2 points), slightly bitter/astringent (2-4 points), bitter/astringent (4-6 points), very bitter/astringent (6-8 points), and extremely bitter/astringent (8-10 points). In the sensory evaluation, sample solution (50 mL) was prepared in a clear glass 30 minutes in advance of the panelists performing the sensory evaluation. First, each panelist sipped ~15 mL of the sample solution and swirled it in the mouth for 7-8 seconds, to evaluate the bitterness, then expelled the solution and evaluated the astringency during the following 3-4 seconds. Finally, each sample was given an overall score. There was a 5-minute interval between the evaluation of each sample and the panelists rinsed their mouths with pure water after each sample evaluation. The evaluations were repeated three times on different days and the results were analyzed statistically.

4. Possible Risks and Discomforts: All samples were of edible grade and did not need to be swallowed into the body and had no side effects on the body.

5. Offer to Answer Questions: If you have any further questions, you can contact the major experimenter Chen yuhong.

6. Signature for Consent: The above-named investigator has answered your questions and you agree to be a research subject in this study. You have carefully read the information and understand fully your rights as a potential subject in a research experiment involving people as subjects.

Subject's Signature: [illegible] Date: 2020.12.25

Investigator's Signature: [illegible] Date: 2020.12.25

Informed consent form

Project Title: Effects of phenolic acids and quercetin-3-O-rutinoside on the bitterness and astringency of green tea infusion

1. Purpose of this Research Study: You have been asked to participate in this study because you are a professional tea sensory reviewer. The aim of this study was to investigate the contribution of phenolic acids and Que-rut to the bitterness and astringency of green tea infusion.

2. Background: Bitterness and astringency are extremely important sensory attributes of green tea infusion, and their intensities strongly influence the desirability and consumption of green tea and related products. Tea polyphenols are very important taste substances in tea infusion, including catechins, flavonols, proanthocyanidins, and phenolic acids, which mainly affect the bitter and astringent taste. There has been research on the bitter and astringent taste of catechins is relatively mature, however, little research has been reported on the sensory evaluation of phenolic acids and Que-rut, and the effects of phenolic acids and Que-rut on the bitterness and astringency of green tea infusion are still unclear.

3. What will be down: You need to score the different monomer substances and tea infusions. The scoring process was carried out in a constant temperature and humidity environment (25°C, relative humidity around 75%). You will be trained to evaluate bitterness and astringency with EGCG solutions of different concentrations and assigned different concentrations of EGCG to the corresponding bitterness and astringency intensity scores. The scoring of taste intensity was done on a 10 points scale, divided into five intervals, i.e., not bitter/astringent (0-2 points), slightly bitter/astringent (2-4 points), bitter/astringent (4-6 points), very bitter/astringent (6-8 points), and extremely bitter/astringent (8-10 points). In the sensory evaluation, sample solution (50 mL) was prepared in a clear glass 30 minutes in advance of the panelists performing the sensory evaluation. First, each panelist sipped ~15 mL of the sample solution and swirled it in the mouth for 7-8 seconds, to evaluate the bitterness, then expelled the solution and evaluated the astringency during the following 3-4 seconds. Finally, each sample was given an overall score. There was a 5-minute interval between the evaluation of each sample and the panelists rinsed their mouths with pure water after each sample evaluation. The evaluations were repeated three times on different days and the results were analyzed statistically.

4. Possible Risks and Discomforts: All samples were of edible grade and did not need to be swallowed into the body and had no side effects on the body.

5. Offer to Answer Questions: If you have any further questions, you can contact the major experimenter Chen yuhong.

6. Signature for Consent: The above-named investigator has answered your questions and you agree to be a research subject in this study. You have carefully read the information and understand fully your rights as a potential subject in a research experiment involving people as subjects.

Subject's Signature: [illegible] Date: 2020. 12. 25

Investigator's Signature: 陈玗宏 Date: 2020. 12.25

Informed consent form

Project Title: Effects of phenolic acids and quercetin-3-O-rutinoside on the bitterness and astringency of green tea infusion

1. Purpose of this Research Study: You have been asked to participate in this study because you are a professional tea sensory reviewer. The aim of this study was to investigate the contribution of phenolic acids and Que-rut to the bitterness and astringency of green tea infusion.

2. Background: Bitterness and astringency are extremely important sensory attributes of green tea infusion, and their intensities strongly influence the desirability and consumption of green tea and related products. Tea polyphenols are very important taste substances in tea infusion, including catechins, flavonols, proanthocyanidins, and phenolic acids, which mainly affect the bitter and astringent taste. There has been research on the bitter and astringent taste of catechins is relatively mature, however, little research has been reported on the sensory evaluation of phenolic acids and Que-rut, and the effects of phenolic acids and Que-rut on the bitterness and astringency of green tea infusion are still unclear.

3. What will be down: You need to score the different monomer substances and tea infusions. The scoring process was carried out in a constant temperature and humidity environment (25°C, relative humidity around 75%). You will be trained to evaluate bitterness and astringency with EGCG solutions of different concentrations and assigned different concentrations of EGCG to the corresponding bitterness and astringency intensity scores. The scoring of taste intensity was done on a 10 points scale, divided into five intervals, i.e., not bitter/astringent (0-2 points), slightly bitter/astringent (2-4 points), bitter/astringent (4-6 points), very bitter/astringent (6-8 points), and extremely bitter/astringent (8-10 points). In the sensory evaluation, sample solution (50 mL) was prepared in a clear glass 30 minutes in advance of the panelists performing the sensory evaluation. First, each panelist sipped ~15 mL of the sample solution and swirled it in the mouth for 7-8 seconds, to evaluate the bitterness, then expelled the solution and evaluated the astringency during the following 3-4 seconds. Finally, each sample was given an overall score. There was a 5-minute interval between the evaluation of each sample and the panelists rinsed their mouths with pure water after each sample evaluation. The evaluations were repeated three times on different days and the results were analyzed statistically.

4. Possible Risks and Discomforts: All samples were of edible grade and did not need to be swallowed into the body and had no side effects on the body.

5. Offer to Answer Questions: If you have any further questions, you can contact the major experimenter Chen yuhong.

6. Signature for Consent: The above-named investigator has answered your questions and you agree to be a research subject in this study. You have carefully read the information and understand fully your rights as a potential subject in a research experiment involving people as subjects.

Subject's Signature: [illegible] Date: 2020.12.25

Investigator's Signature: [illegible] Date: 2020.12.25

Informed consent form

Project Title: Effects of phenolic acids and quercetin-3-O-rutinoside on the bitterness and astringency of green tea infusion

1. Purpose of this Research Study: You have been asked to participate in this study because you are a professional tea sensory reviewer. The aim of this study was to investigate the contribution of phenolic acids and Que-rut to the bitterness and astringency of green tea infusion.

2. Background: Bitterness and astringency are extremely important sensory attributes of green tea infusion, and their intensities strongly influence the desirability and consumption of green tea and related products. Tea polyphenols are very important taste substances in tea infusion, including catechins, flavonols, proanthocyanidins, and phenolic acids, which mainly affect the bitter and astringent taste. There has been research on the bitter and astringent taste of catechins is relatively mature, however, little research has been reported on the sensory evaluation of phenolic acids and Que-rut, and the effects of phenolic acids and Que-rut on the bitterness and astringency of green tea infusion are still unclear.

3. What will be down: You need to score the different monomer substances and tea infusions. The scoring process was carried out in a constant temperature and humidity environment (25°C, relative humidity around 75%). You will be trained to evaluate bitterness and astringency with EGCG solutions of different concentrations and assigned different concentrations of EGCG to the corresponding bitterness and astringency intensity scores. The scoring of taste intensity was done on a 10 points scale, divided into five intervals, i.e., not bitter/astringent (0-2 points), slightly bitter/astringent (2-4 points), bitter/astringent (4-6 points), very bitter/astringent (6-8 points), and extremely bitter/astringent (8-10 points). In the sensory evaluation, sample solution (50 mL) was prepared in a clear glass 30 minutes in advance of the panelists performing the sensory evaluation. First, each panelist sipped ~15 mL of the sample solution and swirled it in the mouth for 7-8 seconds, to evaluate the bitterness, then expelled the solution and evaluated the astringency during the following 3-4 seconds. Finally, each sample was given an overall score. There was a 5-minute interval between the evaluation of each sample and the panelists rinsed their mouths with pure water after each sample evaluation. The evaluations were repeated three times on different days and the results were analyzed statistically.

4. Possible Risks and Discomforts: All samples were of edible grade and did not need to be swallowed into the body and had no side effects on the body.

5. Offer to Answer Questions: If you have any further questions, you can contact the major experimenter Chen yuhong.

6. Signature for Consent: The above-named investigator has answered your questions and you agree to be a research subject in this study. You have carefully read the information and understand fully your rights as a potential subject in a research experiment involving people as subjects.

Subject's Signature: 张燕江 Date: 2020.12.25

Investigator's Signature: 陈[illegible] Date: 2020.12.25
