# Supplementary material for: Effects of phenolic acids and quercetin-3-O-rutinoside on the bitterness and astringency of green tea infusion
Source: NPJ Sci Food. 2022 Jan 27;6:8. doi: 10.1038/s41538-022-00124-8 (PMC8795203; doi:10.1038/s41538-022-00124-8)
Supplement: Supplementary file 3 — The Form of Human Sensory Ethical Committee Inspection [file 41538_2022_124_MOESM3_ESM.pdf]

食品与生物工程学院感官实验伦理审查表

The Form of Human Sensory Ethical Committee Inspection

编号(No)：____________

<table>
<tr><td colspan="3">申请单位（Name of school）：中国农业科学院茶叶研究所茶深加工与多元化利用团队</td></tr>
<tr><td colspan="3">单位地址（Address）：浙江省杭州市梅灵南路 9 号</td></tr>
<tr><td colspan="3">拟申请的项目名称（Study title）：
典型酚酸类化合物的呈味特性及对绿茶茶汤苦涩味的影响
Effects of phenolic acids and quercetin-3-O-rutinoside on the bitterness and astringency of green tea infusion</td></tr>
<tr><td colspan="3">实验目的（Aim of experiment）：按照实验要求邀请具有专业背景的茶叶感官审评员，组成审评小组。基于不同感官评价条件，对审评小组成员进行苦味、涩味的感知训练，系统研究绿茶中的典型酚酸类物质，明确酚酸类以及其他酚类物质对茶汤苦味、涩味的贡献度，探明典型酚酸类物质的呈味特性、酚酸类物质对表没食子儿茶素没食子酸酯（EGCG）呈味的影响以及酚酸类物质对茶汤呈味的影响。</td></tr>
<tr><td colspan="3">实验方法（Experimental methods）:
（1） 感官审评小组由 5 位具有高级评茶员资格的人员组成，包括 3 位男士、2 位女士，年龄范围 25~48 周岁，均为中国农业科学院茶叶研究所的教职工或学生。审评过程在恒温恒湿（25℃，相对湿度 75%左右）的感官审评室中进行。
（2） 试验中对审评小组成员进行苦味、涩味的感知训练，所用训练物为不同浓度的 EGCG 溶液，训练结果显示小组成员均能在审评不同浓度的 EGCG 时，将其正确对应上苦味、涩味强度分值，即展开相关审评实验。
（3） 以同样的步骤制作多种绿茶茶样，分别对不同绿茶茶样感官评价，按照实验设计对感受到的苦味和涩味强度进行判断。每两次评价之间采用苏打饼干和纯净水进行口腔矫味。
（4） 分别配制绿原酸、咖啡酸、没食子酸和芦丁等苦涩味物质的不同浓度梯度的单一溶液。随后，感官审评小组成员对上述味觉物质进行感官评价，按照实验设计对感受到的稳态的和动态的苦味强度和涩味强度进行判断。每两次评价之间采用苏打饼干和纯净水进行口腔矫味。
（5） 分别配制不同浓度的 EGCG 溶液与不同浓度的绿原酸、咖啡酸、没食子酸、芦丁溶液的混合溶液。随后，感官审评小组成员对上述味觉物质进行感官评价，按照实验设计对感受到的稳态的和动态的苦味强度和涩味强度进行判断。每两次评价之间采用苏打饼干和纯净水进行口腔矫味。
（6） 按照 1:50 的茶水比、冲泡时间 4 min 制作绿茶茶汤，并在其中加入不同浓度的绿原酸、咖啡酸、没食子酸、芦丁等苦涩味觉物质。感官评价分析员对上述混合茶汤样品进行感官评价，按照实验设计对感受到的稳态的和动态的甜味强度和酸味强度进行判断。每两次评价之间采用苏打饼干和纯净水进行口腔矫味。</td></tr>
<tr><td rowspan="2">感官实验概况</td><td colspan="2">参与者（Participants）：5</td></tr>
<tr><td>数量　3 男士；2 名女士</td><td>申请日期（Application date）：2020 年 12 月 15 日</td></tr>
</table>

<table>
<tr>
<td>声 明
(Declaration)</td>
<td colspan="5">我将自觉遵守感官实验伦理原则，随时接受委员会的监督与检查，如违反规定，自愿接受处罚。(I will abide by human welfare and ethics principle, ready to accept the Commission of supervision. Otherwise, I will voluntarily accept the punishment.)

项目负责人签名（章）(Signature)：

2020 年 12 月 15 日</td>
</tr>
<tr>
<td rowspan="2">审查结果
(是否同意
申请人的
实验方案)
(Results of inspection)</td>
<td>负责人意见
(Study director)：</td>
<td>☑ 同意
(Agree)</td>
<td>□ 不同意
(Disagree)</td>
<td colspan="2">签 名
(Signature)</td>
</tr>
<tr>
<td>浙江工商大学感官实验伦理委员会意见
(Human Ethics Committee)：</td>
<td>☑ 同意
(Agree)</td>
<td>□ 不同意
(Disagree)</td>
<td colspan="2">签 章
(Stamp)
浙江工商大学
实验动物福利伦理
审查委员会</td>
</tr>
</table>

备注 (Supplement)：
